# General Sessions

**Published:** 1877-07

**Authors:** 


### EXAMINER.

---

Vol. XXXV.—JULY, 1877.—No. 1.

---

### American Medical Association.

### TWENTY-EIGHTH ANNUAL MEETING.

HELD IN THE CITY OF CHICAGO, JUNE 5, 6, 7 & 8, 1877.

Tuesday, June 5.—First Day.

The Association met in Farwell Hall, and was called to order at 11 A. M., by Dr. J. Marion Sims, the retiring President, who, in fitting words, thanked the Association for the honors it had conferred upon him, paid a flattering compliment to the founder of the Association, Dr. N. S. Davis, of Chicago, and, with a glowing reference to the labors of Dr. H. I. Bowditch, of Boston, introduced him as the incoming President.

In response to this pleasant introduction, Dr. Bowditch expressed his pleasure and congratulations to the Association, upon its meeting in the Queen City of the West, under such propitious circumstances; and expressed the hope that all would retire from the meeting, feeling that they possessed good will toward all men, and that they had learned something which might be utilized for the benefit of suffering humanity, and which could be carried with them till they were transferred from this sphere of human activity.

Prayer was then offered by the Rev. William L. Harris, D. D., LL. D., who invoked the blessing of God upon the Association, and its annual meetings.

Upon a call from the President, Dr. N. S. Davis, chairman of the Committee of Arrangements, delivered the address of welcome on the part of the profession in Chicago; referring in a happy manner to the evidences of characteristic enterprise in the city which gave them greeting. He briefly mentioned the institutions which the delegates might desire to visit, as well as the receptions of a social nature to which they were bidden. A touching reference was made to the preliminary meeting, held thirty-one years ago in the city of New York, when organization was first effected. In that meeting were seventy-six voters, of which one alone was then in attendance in Chicago, Dr. W. T. Atlee, of Philadelphia. Nearly all the original number had passed to their final reward. Of the twenty-eight presidents, sixteen had found a last rest from their labors. Reference was made to the death of Dr. Henry F. Askew, of Delaware, an ex-president of the Association.

In closing Dr. Davis again assured his hearers of a most general and cordial welcome from the people of Chicago, and said he hoped they would carry on the work of their profession even more vigorously, more nobly, and more successfully than they had in the past, and that these annual greetings would continue while the country endured, and as long as time should last. [Applause.]

Dr. Norman Bridge, on the part of the Committee of Registration, read the list of delegates whose credentials had been approved, and whose names had been duly registered.

The following gentlemen were elected members by invitation: D. F. Boughton, of Mendota, Wis.; W. H. Bunker, of Cincinnati; J. A. Reed, Dixmont, Pa.; D. Leavitt, and Eichberger, of Terre Haute.

The following gentlemen were elected permanent members: Drs. J. K. Bartlett and E. W. Cross, of Minnesota;

Drs. D. A. K. Steele, S. A. McWilliams, John E. Owens, Charles T. Parkes, E. O. F. Roler, Charles L. Rutter, D. T. Nelson, J. S. Knox, W. E. Quine, W. S. Nevins, M. P. Hatfield, Thomas Bevan, E. W. Sawyer, and L. H. Montgomery, all of Chicago, and S. M. Hamilton, of Monmouth, Ill.

The President invited the retiring President, and also the delegates from the Canadas, to take a seat upon the platform.

PRESIDENT'S ADDRESS.

Dr. Bowditch then delivered the President's annual address, of which the following is a brief abstract:

In accordance with precedent, he desired to touch upon topics tending to improve the practical working of the Association, and would therefore make suggestions on the past, the present and the future.

Prior to 1847, the medical profession of the country, as a united body, did not exist; local societies having been formed here and there, but in consequence of political differences, and the difficulty of communication, medical men were alienated rather than drawn together. The result of the union accomplished, has been made evident in the establishment of valuable friendships, which would otherwise have never been made, in the dropping of idiosyncrasies, and in the rapid and easy coalesence of medical men, immediately after the civil war.

The speaker called attention to the enthusiasm prevalent at the earlier meetings, and deprecated the use of wine, which at times used to flow freely at the public and private gatherings.

Respecting the present status of the Association, the speaker felt sure that the meetings had lost reputation in the Eastern and Middle States—there was a lack of co-operation of the entire profession—prominent Western and Southern men absented themselves—many young men and some scoffing elders considered the body a hindrance to the progress of scientific medicine. Some of the reasons for this were, violent discussions of points of order, or ethics; intemperance, disappointment of these having a high standard of the profession; the *cacoethes scribendi aut loquendi* of some individuals; bulky and valueless transactions, occasionally containing papers which had been previously printed; violent partisanship; the effort to pass resolutions pledging the Association to one side or another of a mooted question; and the democratic principle of its organization by which representation was denied to hospitals and colleges.

The following propositions were made with a view to changes which were much needed:

The sections should perform their critical duty by preventing the transactions from being burdened with papers of small value, and thus not permit writers to fail of the highest standard of scientific attainment. Though the sections have in many directions proved themselves of great value, still he did not believe that the desirable result would ever be obtained by trusting to them alone. To obviate the difficulty, the wise regulations of the Smithsonian Institution were recommended; viz.,

1. The publication of only such papers as, after approval by the sections, shall have been submitted to experts whose names are unknown, and whose decision shall be final.

2. The rule that no paper should be published unless it either (a) contributed something new, or (b) made a valuable analysis or arrangement of facts already known.

The incalculable value of the establishment of the judicial council, in 1873, was pointed out; and the following proposition submitted to them:

The appointment of a standing committee for the purpose of procuring scientific papers for each annual meeting, from the ablest men in the various sections of the country, such papers to have precedence over voluntary papers, which latter were not for that reason to be discouraged. It was suggested that a small committee, of five, for example, could be constituted in such a manner that each member might hold office for five years, one leaving annually. Originally created by the judicial council, this committee might afterward itself fill vacancies, the oldest in service acting as chairman in the last year of his office; one or more of the older members of the profession might be there represented, the majority being made up of earnest, accomplished, middle-aged, or younger men. The committee should hold its sessions, each year, during the week of annual meeting, and should, if possible, select the best men at one meeting, and engage them to prepare papers for the next ensuing. All these papers should be referred to experts after reading, and wide public notice should be given, months before the meetings, of their titles and the names of their authors.

The speaker thought that in two points the Association would do well to follow the lead taken at Louisville, in 1875, (1) in abstaining from all intoxicating drinks, and (2) in extending an invitation to women to meet with members in social intercourse. Even those not believing in the propriety of total abstinence or prohibition, should be willing to make concessions for the sake of the weaker brethren; and such a step taken by the Association would have an indirect but powerful effect on the cause of temperance.

Two other suggestions: (1) Every honorable, well-educated physician in the United States, should become a member of the Association by the very fact of his having become a physician. Every member of a State society should become a permanent member of the Association. (2) The honor of becoming a delegate would be greater, if each society could send one representative only for each 20, or perhaps 30, members; and possibly the best men of the profession would be willing then to accept the honor.

The following reasons were given against the proposed union with the Canada Medical Association: 1, the large dimensions of the American Association; 2, the two languages employed in Canada; 3, the difficulty of arranging for expenses incidental to such union; 4, the distance between places where meetings might be held.

On the other hand, in favor of the plan were: 1, the high standard of the British schools; 2, the evident fact that the large Canadian constituency might send delegates precisely as a new State Medical Society in a distant State; 3, the value of such a union in the way of promoting good-will between the two countries; 4, the opening up of such cities as Montreal and Quebec as places of meeting. He suggested that the whole matter be referred to the judicial council, to report upon the feasibility of union, or of biennal or quinquennial congresses of the two bodies; with the nomination of a committee to act with one chosen by the Canadian Association, the joint committee to draft a plan for submission subsequently to the two bodies.

Proceeding next to the question of the American Pharmacopœia and the relations of the Association to it, the speaker detailed the resolutions proposed, the previous year, by Dr. E. R. Squibb, of Brooklyn, N. Y., and carried by the Association. He then proceeded to sketch the history of the Massachusetts, New York, and Philadelphia Pharmacopœias, and the method by which the last-named which has survived the others, is still published by the Decennial Convention of delegates from incorporated colleges of medicine and pharmacy. The American Medical Association had never taken any action upon the work of the two conventions held since its organization, first, because it was not invited to do so; and, second, because, never having been *incorporated*, it had no right to do so.

Under the circumstances, therefore, the Association might either, (1), procure an act of incorporation before 1879; (2), send delegates to the convention of 1880, and ask for their admission, or, (3), adopt the plan of Dr. Squibb, and appoint a pharmaceutical council, with the co-operation of the army and naval medical men, and the American Pharmaceutical Association.

The complaints against the present pharmacopœia are: first, that it is not sufficiently cosmopolitan; second, that it is too unfrequently published, a demand being made for an annual tentative selection of formulæ, in accordance with the plan followed by the publishers of the German Codex; third, that the pharmacopœia is not really national in authority and scope; finally, that the dispensatory of 1870, fails to meet the edition of the pharmacopœia of that year.

The plan suggested by the Chicago College of Pharmacy involved the selection of two committees—one of physicians, the other of druggists, to divide between them the labor of devising and directing the preparation of formulæ, and of publishing the results—the chief objection stated was the implied limitation of the druggist to the remedies proposed by the physician.

The objections and suggestions stated above could not be applied to the Dispensatory, which was a private work, valuable in character, and remunerative to its copyright holders.

The protests of the Philadelphia County Medical Society, the Philadelphia College of Physicians, and the Philadelphia College of Pharmacy, were laid before the Association—all against any action by the latter in the premises.

In conclusion of this subject, the speaker urged the appointment of a committee to the convention of 1880, with instructions to report upon the result of their inquiries into the matters connected with the pharmacopœia. The presumption to be formed from the not rapid sale of the previous publications of the American Medical Association, did not promise much success in an undertaking of the kind proposed.

The speaker then briefly referred to three important subjects: first, the committees on State Boards of Health, who were doing a good work; second, the influence to be exerted upon members of Congress, relative to the museum and library of the Surgeon General's office, U. S. Army, its need of national support, its great value and usefulness; third, the interest from funds collected to keep alive the memory of the first ovariotomist, Caldwell, and its disposal in premiums, not merely to writers upon the uterus and its appendages, but to those "who have confessedly promoted the welfare of mankind by original conceptions, essays or contributions to medical science." This was considered liberal, but the speaker thought the subject of gynecology in its broadest sense, sufficient to satisfy the desires of all.

The President concluded by saying that the future of the Association depended mainly on the way in which the physicians of this country, and especially the young physicians, did their duty toward it. If the best men in the profession would not come to its meetings and work for the common good of all; if they stood aloof and complained of the inferiority of its work, or actually scoffed or sneered at it, it would accomplish less than it could wish. But it should certainly love to invite to its meetings every prominent physician in the country, and if those who did attend the meetings would determine that nothing but what was excellent should be published in its works, then they would be doing a really noble work, and the Association would eventually claim the highest respect of the whole profession. [Applause.] It was growing stronger every year. It had enjoyed a perpetual youth, a stalwart manhood, and, he sincerely trusted, it would live to a genial old age. [Applause.]

On motion made by Dr. Brodie, of Detroit, the thanks of the Association were returned to its President for his interesting address. The address was referred to the Committee on Publication.

Dr. Brodie further moved that a committee of seven be appointed to report upon the recommendations embraced in the President's address.

The first Vice-President, Dr. N. J. Pittman, of North Carolina, announced the committee, as follows: Dr. W. B. Brodie, of Detroit; Dr. S. D. Gross, of Philadelphia; Dr. E. Grissom, of North Carolina; Dr. J. R. Smith, of U. S. A.; Dr. J. R. Bartlett, of Minnesota; Dr. J. P. White, of Buffalo; Dr. J. M. Toner, of Washington.

A number of papers were then read by title and referred to their appropriate sections; after which the Association adjourned to meet at 9:30 A. M. on Wednesday, June 6, 1877.

---

### Wednesday, June 6.—Second Day.

The Association was called to order at 9:30 A. M. by the President. The minutes of the previous meeting were read and approved. A recess of ten minutes was taken to allow the delegates opportunity to select their representatives for the Nominating Committee, and report such selections to the Secretary.

The following volunteer papers were referred to their appropriate Sections: *On Plastic Splints*, by Dr. H. O. Marcy, of Massachusetts; *On Epithelioma*, by Dr. S. P. Breed; *On a New Speculum*, by Dr. E. A. Hildreth, of West Virginia; *On Conservative Surgery*, by Dr. I. N. Quimby, of New Jersey; *A New Instrument*, exhibited by Dr. H. I. Bowditch, of Boston.

PERMANENT MEMBERS.

Dr. Davis submitted the following names for election as permanent members: Drs. F. C. Schaefer, Chicago; W. H. Fitch, Rockford, Ill.; G. H. Chapman, Hudson, Mich.; G. E. Willard, Chicago; C. C. Hunt, Dixon, Ill.; J. J. Stone, G. Benedict, and J. H. Stewart, of Minnesota; J. N. O.Brien, Plymouth, Wis.; R. Dexter, and J. Vider.

REPORT ON THE PRESIDENT'S ADDRESS.

Dr. Brodie, of Michigan, chairman of the special committee appointed to consider the recommendations embodied in the President's annual address, submitted the following report:

"Your special committee, to whom was referred the recommendations in the President's annual address, respectfully report that they have had the same under consideration, and recommend as follows:

"1. The Smithsonian plan: It is believed that if the officers of the Sections should perform their duties promptly under the existing regulations, there would be no necessity for further examination.

"2. In the matter of a standing committee to procure papers on scientific subjects, it is or should be part of the duty of the chairmen of the Sections to obtain suitable matter for their respective Sections, at as early a time after their appointment as possible; and it is believed this would effect what the President proposes.

"3. On permanent members and representation, we do not think it best at the present time to make or suggest any change in the present plan of organization.

"4. On the union of this Association with the Canada Medical Association, we consider the same impracticable, and are of opinion that the present system of intercourse between the societies by delegates serves to meet the requirement.

"5. On the question of the Pharmacopœia, we deem it inexpedient at the present time to take any action in the premises."

On the suggestion of Dr. Bowditch, the report was laid upon the table temporarily.

REVISION OF THE U. S. PHARMACOPŒIA.

The hour of ten having arrived, Dr. E. R. Squibb, of New York, spoke on the revision of the United States Pharmacopœia. He stated that he was ready to report on this important matter, but saw from the programme that only one hour had been allowed for it. He would be unable to do more than to make brief allusion to the matter in that time, and was unwilling to dispose of it in any such cursory manner. It was deserving of a long and deliberate consideration. Yet he was willing to conform with the wishes of the Association and do as directed.

Dr. Davis, on behalf of the Committee on Arrangements, said that though this subject had been assigned to this hour, it might be laid on the table, at the close of the hour, for further consideration.

It was decided, on motion of Dr. Gallagher, to hear Dr. Squibb to the end of the hour.

The report, as read by Dr. Squibb, involved a full resumé of the arguments *pro* and *con* on the revision of the Pharmacopœia. It involved copious quotations from Dr. Wood's pamphlet on the subject. It denied the allegations of Dr. Wood that it was the intention by the proposed innovations, to abolish the old Pharmacopœia, but asserted that the design was to improve the present plan by introducing new and important revisions similar to those lately made in the German Pharmacopœia. Nor was it designed to interfere with the "Dispensatory," but merely to have a Pharmacopœia without a Dispensatory. It was intended by the new revision simply to undertake a work which the old Pharmacopœia did not do. He explained that it was intended by the revision to make the Pharmacopœia explain its own assertions, or definitions, without the aid of a Dispensatory. He referred to many mistakes in the Dispensatory of 1877, showing little improvement since 1873, and which should be remedied. In refutation of the argument that the whole profession, by the proposed plan, would be under the control of one man, he averred that it should be remembered that that man would be the President of the American Medical Association; and that no more impropriety obtained in that, than in the representation of an entire Government by one Minister, as often occurred in national matters.

The time having arrived for another order of business, the subject was referred to the Committee on Arrangements to appoint an hour for its further consideration.

The next order of business was an

ADDRESS BY THE CHAIRMAN OF THE SECTION IN PRACTICAL MEDICINE, ETC., which was delivered by Dr. P. G. Robinson, of Missouri. The address consisted of a review of the progress made in medicine during the past year. It had been fully shown that accumulations of sewerage and the like became centres of contagion. Especial attention was directed to the etiology of specific fevers, notably the typhoid, and reference was made to the outbreak which occurred in Lancashire, England, in consequence of using milk supplied from one dairy. The attention of the Association was called to an article in the July number of the *American Journal of Medical Sciences*, where the cure of a case of rabies canina by the use of strychnine was reported by Dr. Watson, of Jersey City. In his case there was no aversion to water shown, and for that, and some other reasons, a number of physicians held that it was not a true case of hydrophobia. The great question to be settled was that of diagnosis.

Dr. Robinson then passed to a consideration of the use of salicine and salicylic acid in the treatment of acute rheumatism. These had been used with great advantage in various hospitals, both abroad and at home, and there was no doubt that a means of alleviating this terrible disease had been found.

Reference was made to the value of counting the blood-corpuscles in certain affections, and quotations made from several French authors.

In physiology reference was made to the labors of Ferrier, Fritz and Hitzig, Dalton, Mitchell, Hughlings Jackson, and others, regarding the localization of function in the brain, the function of the corpora quadrigemina, the duality of the vaso-motor system, etc. During the past year several drugs had been brought into use, and special mention was made of the use of gelseminum in the treatment of facial neuralgia, salicylic acid as an internal and external remedy, erythroxylon coca, and several other articles. The address was well received, and referred to the Committee on Publication.

ADDRESS OF THE CHAIRMAN OF THE SECTION ON OBSTETRICS.

Dr. Jas. P. White, of Buffalo, N. Y., delivered the address, and began by saying that the most notable event of the year was the formation of the American Gynæcological Society, and its transactions and publications were of great interest to the medical profession. Dr. White then called attention to a number of books and pamphlets in relation to this particular branch of medical study which were published last year.

Special and favorable mention was made of the writings of the late Dr. John S. Parry, and his book was reviewed to considerable length. The papers of Dr. Isaac E. Taylor, read before the New York Academy of Medicine, received special notice, but he was inclined to differ with Dr. Taylor as to the value of Cæsarean section when compared with craniotomy. The theory of Dr. Maxson, of Syracuse, N. Y., regarding the value of the knee-and-elbow position in the management of cases of malposition of the fœtus, received favorable mention. Pneumatic pressure, by Dr. Campbell, of Georgia, was noticed and believed to possess some value. Dr. White was pleased to notice the growing sentiment in favor of the use of the obstetrical forceps, and exhibited a pair made in accordance with his own views regarding the construction of the instrument. The management of placenta prævia was under consideration when the hour expired.

COMMITTEE ON NOMINATIONS.

The Committee on Nominations was announced as follows:

Dr. J. M. Keller, Arkansas; Dr. L. M. Lovelace, California; Dr. C. R. Bissell, Colorado; Dr. H. M. Knight, Connecticut; Dr. W. Marshal, Delaware; Dr. W. H. Ross, District of Columbia; Dr. R. Battey, Georgia; Dr. C. H. Rawson, Iowa; Dr. T. D. Fitch, Illinois; Dr. G. Sutton, Indiana; Dr. W. L. Schenck, Kansas; Dr. D. W. Yandell, Kentucky; Dr. J. C. Egan, Louisiana; Dr. W. B. Cobb, Maine; Dr. H. O. Marcy, Massachusetts; Dr. C. H. Ohr, Maryland; Dr. L. Connor, Michigan; Dr. C. P. Adams, Minnesota; Dr. T. B. Lester, Missouri; Dr. W. M. Compton, Mississippi; Dr. S. G. Dearborn, New Hampshire; Dr. S. Lilly, New Jersey; Dr. J. P. Gray, New York; Dr. E. Grissom, North Carolina; Dr. W. W. Jones, Ohio; Dr. S. D. Gross, Pennsylvania; Dr. W. H. Palmer, Rhode Island; Dr. W. H. Geddings, South Carolina; Dr. D. J. Roberts, Tennessee; Dr. A. E. Carothers, Texas; Dr. William R. Hutchinson, Vermont; Dr. F. D. Cunningham, Virginia; Dr. J. C. Hupp, West Virginia; Dr. J. T. Reeve, Wisconsin; Dr. J. R. Smith, United States Army.

Dr. Nichols, of the District of Columbia, President of the Association of Superintendents of Insane Asylums of the United States, was then introduced by Dr. N. S. Davis, and the Association adjourned to meet at 9:30 A. M., June 7, 1877.

---

THURSDAY, JUNE 7.—THIRD DAY.

The Association convened at 9:30 a. m., Dr. Bowditch presiding; the attendance was as large as on the previous day. The list of those who had registered was read by the Secretary, the total number of delegates now present being given as 660.

Dr. N. S. Davis reported the following names proposed as members by invitation: Dr. H. P. Godfrey, of Berlin, Wis.; Dr. J. R. Moffatt, Prairie du Chien; Dr. R. G. Floyd, Whitehall, Wis.; Dr. G. F. Everett, Dixon, Ill.; Dr. Truman W. Miller, Chicago; Dr. Thomas R. Douglas, Pennsylvania; Dr. C. M. Fitch, Chicago.

The following were proposed as permanent members: Samuel Tibbets, M. D., Kirkwood, Ill.; G. M. Chamberlain, M. D., Chicago; C. G. Simmons, M. D., Chicago; Charles W. Chaffee, M. D., Chicago; William M. Kaull, M. D., Princeton, Ill.; James H. Wallace, M. D., Monmouth, Ill.; John A. Meek, M. D., Jonesboro, Ind.; Josiah Rogers, M. D., Ripon, Wis.

Both reports were received, and the members declared elected as proposed.

The Secretary stated that the only reports as to necrology were those received from the States of Indiana, Maryland, and Missouri, and from the United States army. He moved the reference of the reports received to the Committee on Publication, which was ordered.

The Committee on Prize Essay reported that but one essay had been placed in its possession in time to permit of a reasonable examination, and that they do not consider it entitled to the prize. Another was received only a few day before the opening of the present session, and, as they had not been able to examine it, they recommended that the author be permitted to withdraw it and present it at the next meeting. The report was adopted.

The Judicial Council reported in regard to several cases before them of disputes as to representation, recommending in the case of the dispute between the Arkansas State Medical Society and the Arkansas State Medical Association, which was decided in favor of the former last year, that no further action be taken, and that the application for the admission of a delegate from the latter body be refused. They also reported favorably on the admission of delegates from the Branch County Medical Society of Michigan, and against the admission of a delegate from the Hendricks County Medical Society. In the matter of certain charges made by Dr. G. E. Frothingham against the Michigan State Medical Society and against Drs. Foster Pratt, of Kalamazoo, and G. K. Johnson, of Grand Rapids, the Committee reported that the charges had no bearing on the professional character of the defendants, and should therefore be dismissed. All the reports were accepted.

The Committee on Publication reported as follows:

Your Committee is constrained to acknowledge that the late date which the volume for 1876 (Vol. XXVII. of the series) was issued needs explanation. The delay of a few months subsequent to the annual meeting is, of course, due to the fact, to which attention has many times been called, that the edition which it is proper to print cannot be arrived at except by means of answers to circulars sent to the members. It is unpleasant to find fault at all, but to be compelled to complain of annually-recurring neglect, if not of downright inefficiency, is an exceedingly ungracious task. The Committee of Publication is profoundly convinced that the proceedings of a medical body like that of the American Medical Association can be adequately reported by men of medical education alone. No skill or training can compensate for the lack of this qualification. And yet for years past the Committee has been annually delayed by a mass of copy prepared by professional reporters, of which a large portion has been undecipherable, and of course unfit to be put into the hands of the printer. We allude to the copy furnished for the minutes of the sections. In this copy medical terms were misspelt in instances without number, while in equally numerous instances gaps represented the words which the reporters were unable to catch or to understand. Sometimes the confusion thus produced was of the most perplexing character. In fact, had it not been for the kind co-operation of several members who had attended the last session of the Association, and who had participated in the discussions, the Committee of Publication would have been forced to eliminate paragraph after paragraph from the minutes. To these causes the delay in the issue of the volume has been principally due, and in view of them the Committee thinks it can consistently claim the indulgence of the Association. The prize essay was put through the press as promptly as the very elaborate character of the work permitted. Of Vol. XXVII., 1,250 copies were printed, 1,207 copies distributed to members, and 43 copies are on hand. Of the prize essay 1,250 copies were printed, of which 1,067 have been distributed, leaving 183 on hand.

The report was referred to the Committee on Publication.

The Treasurer reported as follows:

Under the positive instruction of the Association the prize essay has been published at a cost of some $6,000. This unusual outlay, in addition to the annual volume of transactions, leaves the Treasury in an exhausted condition, as it has practically given to each member $9 in value for $5 received. In this last report, after twenty-two years of service, it remains for the Treasurer to thank the Association for its long-continued confidence, and to regret that in leaving the Treasury solvent he cannot leave a more abundant surplus.

It was stated that the Treasury now contains a balance of $172.72. This report was also referred to the Committee on Publication.

Dr. Erzra M. Hunt, of N. J., Chairman of the Section on State Medicine and Public Hygiene, read a paper in regard to the use of vaccination and inoculation in zymotic diseases. He argued that the good effects shown in the case of small-pox tended to show that there was strong reason to believe it to be applicable to all zymotic diseases. He believed that it was possible, by energetic preventive measures, to practically remove these diseases, and contended for a more efficient sanitary police. If municipalities would furnish means, Boards of Health could go for epidemical disease in as systematic a way as a fire-engine went to a fire, and could as effectually stamp it out. Public hygiene received much greater attention in England than in this country, and engaged the attention of many of her most prominent scientific men. There is no better way to train the medical student than to familiarize him with the methods of the best sanitary records. In conclusion, the lecturer urged upon all medical colleges to pay greater attention to the subject of public hygiene, and to make its study a part of the college curriculum. He believed that in the time to come, by paying the strictest attention to sanitary principles, disease might be almost entirely prevented, and instead of needing two Pharmacopœias they would beable to dispense with any. There was no fear, however, that Othello's occupation would be gone, but the profession would become a preventive force.

The paper, which was well received, was ordered referred to the appropriate Section.

The Secretary read the report of the Librarian, as follows:

During the past year, as shown by the catalogue, there have been added to the library 187 distinct titles, exclusive of yearly volumes of transactions of societies, reports of hospitals, boards of health, and volumes of medical journals, where these have been previously catalogued as distinct titles. This addition makes the library to consist at present of 817 distinct titles, which comprehend from a general estimate about 2,034 volumes, inclusive of pamphlets. In the catalogue will be found the names of forty-six American medical periodicals and nine American, national and State, medical societies, which, agreeably to previous resolutions adopted by this Association, send their publications in exchange for the yearly volume of Transactions. It is to be regretted that the list does not comprise all similar institutions in the United States, but as year by year, as shown by the Treasurer's report, renders previous volumes of the Transactions difficult to obtain, these institutions will no doubt endeavor to secure copies for their libraries in due season. It is exceedingly perplexing to the Librarian to have a State Society, for instance, on ascertaining that it needs a set of the Transactions of this Association, offer to exchange its own publications therefor, and to be obliged to reply that ten out of the twenty-seven annual volumes are out of print, and that it is impossible to supply them. All of these institutions have in previous years been duly notified of the objects, aims, and wants of this library. A change of officers may, in some instances, have caused these notifications to be overlooked or neglected. It is in the department of foreign exchanges that the library is reaping its most valuable benefits. With the number of volumes (100) now allowed yearly for that purpose, in time all foreign institutions which issue publications of interest to medical men, and which desire to exchange, may reasonably be expected to be included in the annual catalogue of additions to the library. In establishing this system, some of the volumes sent out will probably not be honored by a suitable return, but this is unavoidable, and year by year these institutions will be eliminated from the list.

Here follows a catalogue of the books received during the year, and a statement of small expenses incurred amounting to $37.20.

The report was referred for publication.

Dr. E. R. Squibb, of New York, then resumed the reading of his paper on the revision of the United States Pharmacopœia. The advantages of his plan were detailed at great length, and several communications from leading medical authorities were read. By a vote taken on the previous day, Dr. Squibb was limited to twenty minutes in order to allow the opponents of the proposed measure to have a show. On motion, this vote was reconsidered, the lecturer having just about gotten to the beginning of his discourse when his time ran out. The vote was close, but enough to give him another twenty minutes, and he ran merrily along for that time. In summing up the lecturer said that there were two points to consider: first, whether the medical society desired to change its Pharmacopœia or not, and if so, who had the power to decide as to how the revision should be made. The Association had three courses before it. The whole subject might be laid on the table, and this would be most acceptable to the speaker, who would thus be freed from all further responsibility in regard to a much disputed question, the discussion of which had already developed much personal feeling. In the second place, they might prepare a plan and offer it to the profession, allowing it to stand upon its merits. And in the third place, they might appoint through the Nominating Committee, a committee of three to prepare a report for presentation at the meeting next year. He would be glad to listen to discussion, and suggested that it be confined to a consideration of these three points. He moved, therefore, that the whole subject be laid upon the table.

This method of promoting discussions did not exactly meet the views of the audience, and was withdrawn.

NOMINATIONS.

The Chairman of the Committee on nominations offered a partial report, and asked a temporary change in the order for the purpose of receiving it. This was granted, and the Secretary read the following recommendations:

For President, T. G. Richardson, of Louisiana; Vice Presidents, J. P. White, New York; Moses Gunn, Illinois; G. W. Russell, Connecticut; A. Dunlap, Ohio. Chairman of Section of Medicine, Materia-Medica, and Physiology, A. L. Loomis, New York; Secretary J. H. Etheridge, Illinois. Chairman Section of Obstetrics and diseases of Women and Children, E. W. Jencks, Michigan; Secretary, H. O. Marcy, Massachusetts. Chairman Section of Surgery and Anatomy, H. H. Smith, Pennsylvania; Secretary, E. Z. Early, Arkansas. Chairman Section of Medical Jurisprudence, Chemistry and Psychology, W. Kempster, Wisconsin; Secretary, E. A. Hildreth, West Virginia. Chairman Section of State Medicine and Public Hygiene, J. L. Cabell, Virginia; Secretary, A. J. Marsh, New Jersey. Next place of meeting, Buffalo, N. Y. Time, first Tuesday in June, 1878.

The report was unanimously adopted.

Discussion on Dr. Squibbs' paper was then commenced, and Dr. H. C. Wood, of Philadelphia, denied that any pecuniary considerations affected the action of gentlemen on this matter. It was a point of honor with them, and not of money. His uncle, Dr. George B. Wood, one of the earliest Presidents of the Association who was unable to be present on account of failing health and intellect, expressed his belief that the proposed measure meant a disruption and dissolution of the Association. That gentleman did not want the Association to put upon record that he had prostituted his position, and that conventions had been manipulated in order to allow him and Dr. Bache to build up their private fortunes out of the present Pharmacopœia. He did not pretend to speak for the University of Pennsylvania, but expressed his own opinions entirely. He contended that the present system of a decennial convention to revise the Pharmacopœia was a good one, and that no necessity existed for any such change as that proposed. Dr. Squibb had stated that if the Association prepared a Pharmacopœia it would have to do it alone. Another would also be prepared in this case, and that of the Association would have to contend against it.

It would not be a fight between the University of Pennsylvania and the American Medical Association, but between a Pharmacopœia recognized by the body of pharmaceutists and that indorsed by a majority of the Association. The consequence would be that two Pharmacopœias would exist side by side, and to which the law of the survival of the fittest would not apply. One would be used in one part of the Union; the other in another section only. The American Medical Association could not agree even on so simple a matter as the nomenclature of disease, and it was not at all likely that they would be able to agree on such a measure as that proposed by Dr. Squibb. He looked upon the Association as a gathering of medical men for social and scientific intercourse, and not as a despot to dictate what opinions a man should hold.

Dr. Brodie, of Michigan, offered the following resolution:

*Resolved*, That a committee of five be appointed by the President, to whom shall be referred the paper of Dr. Squibb, and all the papers on the subject of the Pharmacopœia, with full authority to examine into the whole question as to the propriety of this Association being a factor in whole or in part in its publication, and report at the next annual meeting.

Dr. N. S. Davis believed that if the Association entered as a factor with any other body in the preparation of a Pharmacopœia, it would have to assume whatever of praise or odium attached to that work. While yielding to none in his respect for the Association, he held that they should keep free from incurring pecuniary obligations, from purchasing copywright interests, and contracting entangling alliances. He believed in allowing the Convention to do its work, and to send such men to that Convention as it could depend on. He favored the indefinite postponement of the whole matter, and made a motion accordingly.

Dr. Brodie withdrew his resolution, and the motion of Dr. Davis was at once put to a vote and carried by a large majority.

The Association then adjourned till 9:30 a. m. next day.

FRIDAY, JUNE 8, FOURTH DAY.

After opening routine business, C. M. Fitch, M. D., of Chicago, was elected a member of the association by invitation; and Drs. L. H. Chew, of Naperville, Ill., and S. O. Ritchie, of Chicago, were elected permanent members.

Dr. Atkinson, the Secretary, presented a list of charges against the Michigan State Medical Society, the import whereof was that it had aided and abetted the Michigan University in graduating scholars in an irregular and exclusive medical course of study. Referred to Judicial Council.

He submitted also the report of the Permanent Secretary, made in conformity with an order issued in 1875, on State Boards of Health, showing that circulars had been forwarded to the Executive of each State in which a health board had not already been organized, urging the need and utility of such boards, and soliciting that the subject be brought before the legislature; that State Boards now exist in Alabama, California, Colorado, Georgia, Illinois, Louisiana, Maryland, Massachusetts, Minnesota, Mississippi, North Carolina, New Jersey, Tennessee, Virginia and Wisconsin, and that resolutions had been introduced into other State Legislatures to effect the establishment of boards; and suggesting that the committee be continued.

This report was ordered to be entered upon the minutes.

The Secretary also read a report by Dr. Seguin, of New York, on the subject of uniformity in medical observation and record, showing the paramount need that existed for such uniformity, and recommending that the association send delegates to the convention of 1880, who should advocate the devisement and institution of a plan to secure more systematic operation in that matter. Received and entered.

The Secretary moved that Thomas M. Dysdale, of Philadelphia, be appointed a delegate to foreign medical associations; and that Clifton E. King, of Boston, be appointed likewise to the Canada Association. These gentlemen were so appointed.

The minutes of the Judicial Council were entered without reading.

Reports from the Chairman of the Sections on Obstetrics,

State Medicine and Public Hygiene, Surgery and Anatomy, Medical Jurisprudence, and Practice of Medicine were duly submitted by the Secretary and referred to the Committee of Publication.

The Nominating Committe presented the following names for officers, and they were elected:

*Assistant Secretary*—E. W. Brush.

*Committee of Arrangement*—T. F. Rochester, J. F. Miner, E. R. Barnes, C. C. Wyckoff, M. B. Folnele, W. C. Phelps, E. W. Brush, all of New York.

*Committee on Publication*—W. B. Atkinson, F. M. Drysdale, A. Fricke, S. D. Gross, C. Wister, R. J. Dunglison, of Pennsylvania; W. Lee, of the District of Columbia.

*Treasurer*—R. J. Dunglison, of Pennsylvania.

*Librarian*—W. Lee, District of Columbia.

*Committee on Library*—Johnson Elliott, District of Columbia.

*Members of Section on State Medicine and Public Hygiene*—J. Cochrane, Alabama; A. M. Carrigan, Arkansas; W. F. Cheney, California; C. Denison, Colorado; C. A. Lindsley, Connecticut; W. Marshall, Delaware; T. Antisell, District of Columbia; J. P. Logan, Georgia; H. A. Johnson, Illinois; T. M. Stevens, Indiana; A. G. Field, Iowa; D. W. Stremont, Kansas; S. Brandles, Kentucky; S. M. Berriss, Louisiana; E. F. Sawyer, Maine; C. H. Ohr, Maryland; H. J. Bowditch, Massachusetts; H. P. Baker, Michigan; C. M. Hewitt, Minnesota; W. Johnstone, Mississippi; J. W. Trader, Missouri; M. W. Russell, New Hampshire; E. M. Hunt, New Jersey; E. Harris, New York; J. Comegys, Ohio; B. Lee, Pennsylvania; E. M. Snow, Rhode Island; R. A. Kinloch, South Carolina; T. A. Achison, Tennessee; A. E. Carothers, Texas; J. L. Cabell, Virginia; L. C. Butler, Vermont; J. Frissell, West Virginia; E. S. Griffith, Wisconsin; J. W. Betton, Florida; C. J. O'Hogan, North Carolina; J. S. Billings, United States Army; J. Wilson, United States Navy.

*Committee on Necrology*—J. M. Toner, District of Columbia, Chairman; W. H. Ross, District of Columbia, Secretary; J. W. Barclay, Alabama; T. E. Merrell, Arkansas; M. Baker, California; G. W. Russell, Connecticut; L. P. Bush, Delaware; W. W. Johnson, District of Columbia; T. S. Hopkins, Georgia; J. H. Hollister, Illinois; J. Moffit, Indiana; S. B. Thrall, Iowa; L. P. Yandell, Kentucky; S. C. Gordon, Maine; A. L. Norris, Massachuseets; D. J. McKerr, Maryland; W. W. Breakey, Michigan; E. C. Cross, Minnesota; A. J. Steele, Missouri; J. Braine, New Jersey; N. J. Pittman, North Carolina; J. Jones, Louisiana; G. M. Smith, New York; G. Mitchell, Ohio; W. C. Warriner, Oregon; H. C. Wood, Pennsylvania; C. W. Parsons, Rhode Island; A. N. Talley, South Carolina; J. B. Lindsley, Tennessee; J. H. Stalnmaker, Texas; W. D. Hooper, Virginia; D. Mason, Wisconsin; O. F. Fassett, Vermont; P. F. Whitehead, Mississippi; W. L. Schenck, Kansas; L. G. Hill, New Hampshire; R. W. Haslitt, West Virginia; J. J. Woodward, United States Army; J. Wilson, United States Navy.

*Judicial Council*—J. K. Bartlett, Wisconsin: T. F. Staples, Minnesota; E. Grissom, North Carolina; W. F. Robertson, S. Lilly, New Jersey; W. M. McPheeters, Missouri; A. T. Woodward, Vermont. The foregoing in place of seven whose terms expire by limitation. P. O. Hooper, of Arkansas, in place of A. Dunlap, whose term expires in 1878.

*Committee on Prize Essays*—E. M. Moore, F. Lothrop, W. Miner, H. R Hopkins, E. W. Dean.

The above were in addition to those elected on the previous day.

Dr. T. D. Fitch moved to amend in Section 2, the article relating to permanent members by striking out, "*and such other members as may receive the appointment by unanimous vote.*" The amendment went over under the rules.

A resolution was also offered through the Secretary to create a section on Ophthalmology, Otology and Laryngology. It was laid on the table until the next meeting.

Dr. N. S. Davis moved the following amendments to the By-laws which lie upon the table until the next annual meeting:

1st. Strike out the whole of the paragraph on page 680 of the Volume of Transactions for 1876, which paragraph commences with "Papers appropriate to the several Sections" etc.

Reasons.—The provision is impracticable and in conflict with the duties of the Committee of Arrangements as specified in the Constitution.

2d. Strike out the third paragraph of Section VIII., and the second paragraph of Section IX., page 683, both relating to medical colleges.

Reason.—Both are obsolete and useless, as medical colleges are no longer represented in the Association.

The report of Drs. J. J. Woodward, U. S. N., and Edouard Seguin, on the progress of uniformity in the means of observation and record of physic, was submitted as follows:

"During your last session you have voted that the question of uniformity in the means of medical observation and of medical records should be presented in your name to the International Medical Congress soon to meet in Philadelphia. This Congress received your communication with an interest enhanced by the warm recommendation of its illustrious President, Prof. Samuel D. Gross, in his inaugural address; and the International Medical Congress, in its turn, voted the sending of delegates to the next International Medical Congress, which is to meet in Geneva, September 1877, with the special commission of pleading there the cause of medical uniformity. Such has been the official progress of this question since your last meeting.

Its technological progress consists (a) in the perfecting and cheapening of the sphygmograph, but not yet to the point of making it as popular as the thermometer. (b) In the reduction of urinometers and microscopes to a uniform standard and to pocket size. (c) In the invention of simple colorimeter and globulimeters, which separately realize the hopes induced fifteen years ago by the brilliant invention of Prof. Mantagazza. (d) The more important conquest of this year has been the acceptance of the metric system by the New York State Medical Society.

It may not be out of place to add that the demand for instruments of positive observation has more than decupled in the last ten years, and that the use of uniform records of medical observation has increased from a few hundreds to thousands annually; a double progress entirely due to the initiative of this great progressive body, the American Medical Association.

During the same period the pharmacists have made parallel efforts to bring uniformity in the products of their trade, which is an accessory to our art; and we must acknowledge that they are somewhat ahead of us. They will be strongly represented in the International Medical Congress of Geneva, and it would be more creditable for them than for us, were they the first to agree upon the terms of this long longed-for uniformity on our own ground. Pharmacy would gain nothing and physic would lose much by leaving disconnected two movements which tend to establish uniformity in the pharmacopœia and in the practice of physic; both uniformities being twin sisters from the same spirit, the aspiration of the human mind toward the next synthesis.

Therefore your reporters on this subject propose that the American Medical Association send special delegates to the International Medical Congress at Geneva—as it did so effectively to the International Medical Congress of Brussels, in 1875—to advocate the adoption of a progressive uniformity of means of medical observation and records, with the concurrence, if possible, of the members of this Congress who will be found there engaged in advocating the application of uniformity in this and other departments of science."

The Association adopted the conclusions of this report in its last general meeting, and fused the delegation to that effect with the other delegation to the permanent medical societies of Europe for 1877-'78, naming Dr. Edouard Seguin, of New York, President of this mixed delegation.

Dr. J. W. Singleton, of Paducah, Ky., offered the following in behalf of the Kentucky State Medical Society:

WHEREAS, It has come to the knowledge of the members of the American Medical Association that a bill known as the Morrison bill for the discontinuance of the tariff on quinine is, at this time, before the Committee on Ways and Means of the Congress of the United States; and

WHEREAS, The welfare of a large proportion of the people in the Western States and Territories, is concerned in the issue of this bill, as well as any movement which will enable them to obtain quinine at a less cost than the enormous prices now paid by the consumer; and

WHEREAS, The opposition to this bill set forth by the manufacturers and trade, does not represent the desire of those who are engaged in the relief of suffering and want, but ignores entirely the necessities of a large population, many of whom are engaged in cultivating the soil and opening up new resources of wealth to the Government in malarial districts; and

WHEREAS, Principles of justice and humanity alike demand free quinine and an open market for the competition of European manufactures; therefore, be it

*Resolved*, That the American Medical Association approve the passage of the Morrison bill for the repeal of the tariff on quinine, and respectfully insist that said bill shall become a law.

*Resolved*, That the Permanent Secretary of this Association be required to transmit the foregoing preamble and resolutions to the Chairman of the Committee of Ways and Means of the Congress of the United States.

The resolutions were adopted by a unanimous vote amid applause.

A resolution was passed expressive of the satisfaction the association feels at the unanimity fast coming to exist between the profession in the North and South.

Dr. Lewis A. Sayre was appointed a delegate to the British Medical Society.

Thanks were rendered to Francis Gurney Smith, for his efficiency in transacting the duties of Chairman of the Committee of Publication, from which position he has retired, after many years' service,

Dr. Bowditch having temporarily retired from the chair, offered the following resolution:

*Resolved*, That the association recommend to the chairmen of the sections, at any place at which we may hereafter meet, the propriety of obtaining from our ablest associates in various parts of the country, papers to be presented to the sections, and that due notice of the names of the writers be given in the medical journals before the meeting.

He moved also that all papers to be published be submitted to the examination of experts whose names should be unknown, and only the most worthy should be published.

Dr. Woodward, of Washington, then offered the following resolution:

*Resolved*, That from this meeting and hereafter, the practice of printing in the transactions of this association the so-called verbatim reports of the debates in the sections, be limited to the papers presented and recommended by the sections for publication, and such as may be actually read and offered by each section during the session of the convention.

On motion of Dr. James P. White, of Buffalo, the entire matter was referred to a committee of five to report at the next annual meeting, of which committee Dr. Bowditch and Dr. N. S. Davis were to constitute two members.

Dr. Bodie, of Michigan, submitted a series of resolutions thanking the committee of arrangements for their liberal dealings with visiting doctors, and for their excellent programme; the people of Chicago at large for their hospitality, especially those who gave receptions to the delegates; the Northwestern and other railroads for their many courtesies, and so forth. They were adopted.

Dr. Squibb asked leave to withdraw his report on the pharmacopœia, which was allowed.

President Bowditch then invited the President-elect, T. G. Richardson, of Louisiana, to ascend the platform, and delivered his retiring address. He thanked the association for the distinction it had conferred upon him the past year. He closed his remarks by congratulating the society upon its new President, and extending, as he said, the hand of Massachusetts to Louisiana, thus signifying the obliteration of all sectional ill feelings.

The act was greeted with a burst of applause, and Dr. Richardson responded in becoming terms, frequently interrupted by applause. As he observed that God was to be thanked that we had now become a national family of States, the expression of concurrence by the delegates was earnest and protracted. Though he could not hope to fill the place of his worthy predecessor, yet he hoped that from the shreds of his incapability a cloak of charity might be woven to cover his faults. He thanked the association for the distinction he had received.

The association then adjourned to meet in Buffalo, N. Y., the first Tuesday in June, 1878.

